# Genome-Wide Association Study (GWAS) for Growth Rate and Age at Sexual Maturation in Atlantic Salmon (*Salmo salar*)

**DOI:** 10.1371/journal.pone.0119730

**Published:** 2015-03-10

**Authors:** Alejandro P. Gutierrez, José M. Yáñez, Steve Fukui, Bruce Swift, William S. Davidson

**Affiliations:** 1 Department of Molecular Biology and Biochemistry, Simon Fraser University, Burnaby, British Columbia, Canada; 2 Faculty of Veterinary and Animal Sciences, University of Chile, Santiago, Chile; 3 Cermaq Canada, Campbell River, British Columbia, Canada; 4 TRI-GEN Fish Improvement Ltd., Agassiz, British Columbia, Canada; China Agricultrual University, CHINA

## Abstract

Early sexual maturation is considered a serious drawback for Atlantic salmon aquaculture as it retards growth, increases production times and affects flesh quality. Although both growth and sexual maturation are thought to be complex processes controlled by several genetic and environmental factors, selection for these traits has been continuously accomplished since the beginning of Atlantic salmon selective breeding programs. In this genome-wide association study (GWAS) we used a 6.5K single-nucleotide polymorphism (SNP) array to genotype ∼480 individuals from the Cermaq Canada broodstock program and search for SNPs associated with growth and age at sexual maturation. Using a mixed model approach we identified markers showing a significant association with growth, grilsing (early sexual maturation) and late sexual maturation. The most significant associations were found for grilsing, with markers located in Ssa10, Ssa02, Ssa13, Ssa25 and Ssa12, and for late maturation with markers located in Ssa28, Ssa01 and Ssa21. A lower level of association was detected with growth on Ssa13. Candidate genes, which were linked to these genetic markers, were identified and some of them show a direct relationship with developmental processes, especially for those in association with sexual maturation. However, the relatively low power to detect genetic markers associated with growth (days to 5 kg) in this GWAS indicates the need to use a higher density SNP array in order to overcome the low levels of linkage disequilibrium observed in Atlantic salmon before the information can be incorporated into a selective breeding program.

## Introduction

Growth and age at sexual maturation are among the most important economic traits in Atlantic salmon (*Salmo salar*) aquaculture, with fish having been continuously selected for these traits since the beginning of selective breeding programs in Norway in the 1970s [1–3]. Early sexual maturation is considered a serious drawback for aquaculture as it retards growth for several months while affecting flesh quality and overall production times [4,5]. Late maturation on the other hand, is a desirable trait in many fish breeding programs because it can lead to larger body size and higher fecundity [6–8]; however, late maturation also means longer generation times, which could be disadvantageous for aquaculture production where short generation intervals are considered to be beneficial since increased growth rate and decreased age at maturity allows farm animal producers to shorten a production cycle. These traits, growth and age at sexual maturation, are complex physiological processes controlled by genetic and environmental factors, including qualitative and quantitative aspects of nutritional availability, behavioral interactions conditioned by intra- and inter-specific demographics and seasonal changes [9,10]. The effect of multiple factors on these traits and their heritable variation between and within populations of Atlantic salmon has been described by Garcia de Leaniz et al. [11]. In spite of the high number of environmental factors that may directly or indirectly influence growth, the heritabilities of growth and age at maturity have been reported to show moderate levels in most cases [2,3,11–15]. This scenario makes artificial selection for these traits plausible, which allows their improvement by means of selective breeding.

Advances in genomic research have significantly improved the tools available for the study of commercially important traits using quantitative trait loci (QTL) analyses. The use of molecular markers spread across the genome and the effects of allele segregation are employed to determine the number, positions and the magnitude of the QTL affecting a trait by statistical associations between marker genotype and particular trait phenotypes. The ability to determine the chromosomal regions that affect economically important traits has led to the implementation of selective breeding based on genetic selection practices by identifying animals with favorable genotypes. In Atlantic salmon aquaculture, marker assisted selection (MAS) could be a valuable addition to current selective breeding programs by improving the accuracy of selection, and therefore the genetic gain [16]. This is particularly true for those traits like disease resistance, flesh quality and pigment uptake that are difficult to measure on the actual broodstock [17]. In this regard, a practical example of MAS in Atlantic salmon is the use of a QTL explaining a high proportion of the total variance for resistance against Infectious Pancreatic Necrosis virus [18–20], which is being implemented into commercial breeding programs.

In the past decades, QTL in salmonids have been mainly detected by means of linkage mapping methods using a relatively small number of microsatellite markers distributed across the genome. Consequently, most identified QTL span a large chromosomal region, from which the identification of a causative gene(s) is problematic. Several studies have described QTL associated with growth and/or sexual maturation in salmonid species including Atlantic salmon [21–25], rainbow trout [26–29], coho salmon [30,31] and Arctic charr [24,32]. Yet, information regarding candidate genes located in these QTL regions is scarce for most species. In rainbow trout, however, a Clock gene was localized in a strong spawning time QTL [33]. Previous studies carried out in other salmonid species like rainbow trout and Arctic charr have shown an apparent link between QTL for sexual maturation and growth [24,26,28]; however, recent evidence suggests that in Atlantic salmon these traits are independent of one another [34].

With the emergence of new sequencing technologies it is possible to obtain thousands of single nucleotide polymorphism (SNP) markers for population genotyping, which has allowed the construction of high density genetic maps [35]. The current SNP array technologies provide better tools for the identification of QTL and specific markers associated with traits of interest than was possible using microsatellite markers. For Atlantic salmon in particular, a 6.5K SNP was developed [36,37] resulting in a SNP linkage map with ∼ 5,500 markers [38]. We previously made use of this SNP array to conduct QTL analyses for body weight at different times during a production cycle of Atlantic salmon [39] and subsequently for age at sexual maturation [34]. We were curious to determine if similar results (i.e., genomic locations and specific genetic markers) would be obtained using a different statistical approach, namely a genome-wide association study (GWAS). GWAS evaluates the association of genetic markers with a trait relying on the levels of linkage disequilibrium (LD) between the markers and the genetic variation affecting the trait, testing for association of each SNP and therefore, making possible the identification of specific alleles associated with the trait.

Here, we present the results of our GWAS in the Cermaq (formerly Mainstream) Canada Atlantic salmon broodstock population making use of the Atlantic salmon 6.5K SNP array [36,37]. We analyzed growth in this commercial population in terms of days to reach 5 kg and also age at sexual maturation. As GWAS does not rely on associations within a family as QTL analysis does, we were able to increase the power to detect association between markers and traits by including an additional 192 Atlantic salmon in all analyses and an additional 160 samples (80 grilse and 80 normally maturing Atlantic salmon) for the grilsing GWAS. To our knowledge, the present study represents the first GWAS for growth and age at sexual maturation in a salmonid species.

## Materials and Methods

### Samples and phenotype data

At this time the Canadian Council on Animal Care does not have formal guidelines for the use of DNA obtained from tissues. Therefore, for the purposes of this research an Animal Care permit from the University Animal Care Committee at Simon Fraser University was not required. Samples came from a commercial breeding program developed by Cermaq (formerly Mainstream) Canada and based on the Mowi strain of Atlantic salmon, which was derived from a breeding program established using Norwegian populations [40]. In November/December 2005, 130 single pair mating families were established. At the fry stage (February 2006) 120 offspring from each family were pooled (15,600 fish in total) and grown communally at the Oceans Farms Hatchery, Vancouver Island. In September/October 2006, 5,000 of the fish were PIT (passive integrated transponder) tagged and phenotypic measurements were taken until early 2009. DNA extracted from fin clips allowed the posterior parental assignment by the use of microsatellites [41]. Five full-sib families comprising 279 individuals (including parents), were selected for analysis. The five families named F007, F023, F076, F088 and F107 contained 51, 62, 45, 46 and 65 progeny, respectively [39].We included an additional 192 Atlantic salmon parents from the same broodstock year (BY) 2005 in all of the analyses. For the grilsing analysis, we also included an additional 160 samples (80 grilse/80 normally maturing Atlantic salmon).

Body weight measurements were taken as described in our previous analysis [39]. Based on the weight measurements taken at times during the production cycle, the number of days required to reach a market weight of 5 kg was calculated for all fish. Maturation times were classified as: precocious (≤ 12 months of age), grilse (36 months of age, at 1^st^ sea winter (SW)), normally maturing (48 to 60 months of age at second SW or third SW), and late-maturing fish (>60 months). For our analyses, maturity was defined as a binary trait in which grilse were recorded as 1 and normally maturing fish were recorded as 0; for the late maturation analysis, fish maturing after 60 months were recorded as 1 and those normally maturing as 0. Sex was recorded during the confirmation of gonad maturation status for fish maturing up to 60 months old. The sex of late maturing fish was determined by the presence of the sdY gene according to Eisbrenner et al. [42].

### SNP array and linkage mapping

The SNP data used for this analysis have been described previously [39]. Five families were selected for SNP genotyping at CIGENE, Norwegian University of Life Sciences, Ås, Norway using an Atlantic salmon 6.5K Illumina iSelect SNP-array [36,37]. Analyses were based on an Atlantic salmon linkage map, which contains ∼5,650 SNP markers and was constructed using genotyping data from 143 families comprising 3,297 fish [38]. This map contains 29 linkage groups, which were assigned to their specific chromosome number according to the nomenclature established by Phillips *et al*. [43]. A total of 471 fish were genotyped for the GWAS. Quality control (QC) was performed and those markers with a call rate lower than 95% and a minimum allele frequency lower than 0.05 were filtered and excluded from the analysis.

### Genome-wide association study

GWAS was carried out using the GenABEL library implemented in R statistical environment (http://www.r-project.org). Considering the presence of closely related fish in our sample, we used the GRAMMAS approach (Genome-wide association using Mixed Model and Score test) [44,45]. Thus, we used the “polygenic” function [46] to fit three different univariate additive polygenic models which were defined by means of the following general formula:

Y=Xb+Sa+Zu+e

where *Y* is the vector of phenotypic records (days to 5 kg, late maturation and grilsing); *b* is the vector of fixed effects (sex for days to 5 kg and late maturation); *a* is the fixed effect of the SNP genotype; *u* is the random additive genetic effect; *X* and *Z* are design matrices for *b* and *u*, respectively; *S* is the design vector for *a*; and *e* is the vector of random residuals. For the three models, *a* and *e* were assumed to be $∼N\left(0,A\sigma_{a}^{2}\right)$ and $∼N\left(0,I\sigma_{e}^{2}\right)$, respectively, where *A* is the additive genomic kinship matrix, $\sigma_{a}^{2}$ is the polygenic additive variance, *I* is an identity matrix and $\sigma_{e}^{2}$ is the residual variance.

In order to take the relatedness between individuals into account by means of a (co)variance matrix, the kinship matrix **A** was calculated using genomic data. The genomic kinship matrix **A** was estimated using marker data with the *ibs* (identity by state) function and *weight = freq* option of GenABEL. The residuals obtained from the model were used to perform an association test by means of a simple least squares method [45–47]. Genome-wide significance was assessed by the use of two different methods: first, empirically using 200 permutations and markers with p-values ≤ 0.05 were considered to be genome-wide significant, and second, by the Bonferroni method, in which the conventional p-value was divided by the number of tests performed. A SNP was considered to have genome-wide significance at p < 0.05/N and have chromosome-wide significance at p < 0.05/Nc, where N is the total number of SNPs used in our study and Nc is the number of SNPs in a particular chromosome.

### Linkage disequilibrium

The levels of linkage disequilibrium as r^2^ were calculated using the GenABEL package, in order to assess the ability of the available SNPs to capture the genetic variation of the traits analyzed. LD was calculated for all adjacent marker pairs using all of the parents in the population to avoid LD inflation by extremely related individuals present in the full-sib groups of the progeny. The extent and decay of LD with distance was analyzed based on the methodology described by Heifetz et al. [48]. Briefly, the equation of Sved [49], which relates LD caused by drift to inter-marker distance and effective population size, was used to summarize the extent and decay of LD with distance:

$$LD_{i}=1/\left(1+4bd_{i}\right)+e_{i}$$

where *LD_i_* is the observed LD for marker pair *i*, *d_i_* is the distance in cM for marker pair *i*, *b* is the coefficient that describes the decay of LD with distance, and *e_i_* is a random residual. Parameter *b* was estimated using nonlinear regression analysis.

### Candidate genes

The nucleotide sequences corresponding to the SNPs that showed a significant association with growth or age at sexual maturation were compared by BLAST against the first assembly of the Atlantic salmon genome sequencing project [50], which is publicly available at ASalBase (www.asalbase.org) and NCBI (http://www.ncbi.nlm.nih.gov/Traces/wgs/?val=AGKD). SNP markers were then assigned to a specific whole genome shotgun (WGS) contig by sequence similarity searches. WGS contigs were annotated using an in-house annotation pipeline (trutta.mbb.sfu.ca).

## Results

A total of 466 samples and 3,908 markers passed the QC and consequently a GWAS was carried out to identify markers significantly associated with growth (in terms of days to 5 kg) and late sexual maturation in Atlantic salmon. In the case of the grilsing analysis, 3,873 markers and 626 samples passed the QC.

### Growth association analysis (days to 5 kg)

**Fig 1:**
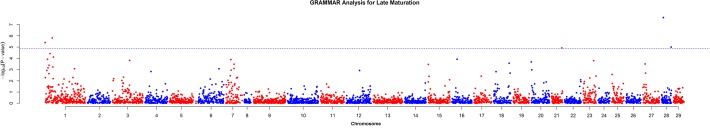
Results from GWAS for days to 5 kg. Horizontal dotted line represents the genome-wide significant threshold.

Only one marker (GCR_cBin15343_Ctg1_36) located on chromosome 13 (Ssa13) was found to be significantly associated (according to the empirical method) with growth (see Fig. 1), but only at the chromosome-wide level of significance (p < 2.55e-4 for Ssa13) according to the Bonferroni threshold. Marker GCR_cBin15343_Ctg1_36, localized in Ssa13 was assigned to the Atlantic salmon genome contig AGKD01005773 and annotation of this contig showed that the marker is located in a hypothetical protein in the vicinity of a membrane-associated guanylate kinase protein as shown in Table 1.

**Table 1:** Days to 5 kg association detected in the analysis.

SNP	Chr^a^	Position (cM)^b^	Alleles	N	Contig^c^	Harbouring gene	Nearest gene^d^	p-value GR^e^	p-value PR^f^
*GCR_cBin15343_Ctg1_36	13	41.8	A/G	395	AGKD01005773	Uncharacterized	MAGI-1	6.31E-05*	0.15
ESTV_20925_1105	4	77.3	A/C	394	AGKD01068721	NPM1	TRN1	8.19E-04	0.74
ESTNV_36199_1181	19	55.6	A/C	389	AGKD01274623	Uncharacterized	DNAH	1.40E-03	0.87
ESTV_14490_591	15	76	A/G	395	AGKD01042308	PTGES3	MIP	1.46E-03	0.88
GCR_cBin1445_Ctg1_135	19	55.6	A/C	395	AGKD01045399	Uncharacterized	DNAH	1.62E-03	0.90
ESTV_16731_596	13	52.3	A/C	395	AGKD01001948	SVEP1	ANKS1A	1.68E-03	0.91

*Marker showing a chromosome-wide significance

^a^ Chromosome number assigned according to Phillips et al. [43].

^b^ Positions obtained from female SNP map described by Lien et al. [38].

^c^ Contig containing the marker sequence as result of BLAST alignment on www.asalbase.org.

^d^ The closest identified gene on the contig, according to BLASTx or BLASTn results.

^e^ p-value obtained from GRAMMAR analysis and subject to Bonferroni significance thresholds.

^f^ p-value obtained from genome wide permutation analysis.

### Sexual maturation association analysis

**Fig 2:**
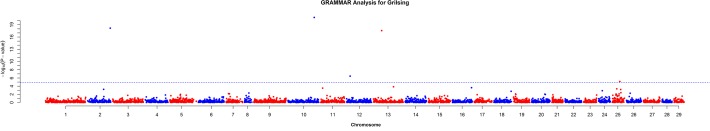
Results from GWAS for grilsing. Horizontal dotted line represents the genome-wide significant threshold.

Analysis for grilsing identified five markers with a genome-wide significant association (p < 1.29e-5 according to the Bonferroni threshold and p < 0.05 for the permutation method) with the trait as shown in Table 2. These markers (ESTNV_20578_482, ESTNV_36582_634, ESTNV_34243_316, GCR_cBin47052_Ctg1_234, ESTNV_15175_311) are located in Ssa10, Ssa02, Ssa13, Ssa25 and Ssa12, respectively (Fig. 2). The most significantly associated marker ESTNV_20578_482 is located within an E2F Transcription Factor (E2F) and nearby the CCR4-NOT transcription complex gene. The next most significant marker ESTNV_36582_634 is located within a malate dehydrogenase gene (MDH) and close to a UDP-glucose pyrophosphorylase gene (UGP). The next marker showing a significant association was ESTNV_34243_316, a SNP located within a PQ Loop Repeat Containing gene. The other two markers that showed a genome-wide significant association with grilsing were located in uncharacterized genes, as shown in Table 2.

**Table 2:** Grilsing association detected in the analysis.

SNP	Chr^a^	Position (cM)^b^	Alleles	N	Contig^c^	Harbouring gene	Nearest gene^d^	p-value GR^e^	p-value PR^f^
**ESTNV_20578_482	10	84.4	A/G	573	AGKD01058461	E2F4	CCR4-NOT	5.09E-20**	0.005**
**ESTNV_36582_634	2	62.7	G/A	585	AGKD01209317	MDH	UGP	1.03E-18**	0.005**
**ESTNV_34243_316	13	50.4	C/A	585	AGKD01027215	PQLC2	NR1H4	3.96E-18**	0.005**
**GCR_cBin47052_Ctg1_234	25	47.7	C/A	585	AGKD01042192	Uncharacterized	NDF1	2.75E-06**	0.005**
**ESTNV_15175_311	12	63.1	A/C	567	AGKD01014695	Uncharacterized	Unknown	8.57E-06**	0.025**
ESTNV_36261_377	13	71.6	A/G	568	AGKD01241736	PGRC1	COX7B	2.56E-04	0.43
GCR_hBin3730_Ctg1_424	16	61.8	G/A	585	AGKD01013005	SYAP1	IFI44	3.33E-04	0.53
GCR_cBin6804_Ctg1_99	2	86.2	A/G	585	AGKD01100126	Uncharacterized	SMAP1	3.99E-04	0.56
ESTNV_30600_327	11	12.9	C/A	584	AGKD01020938	Uncharacterized	MYPC	5.41E-04	0.65
GCR_cBin43301_Ctg1_366	25	34.3	G/A	585	AGKD01168272	Uncharacterized	PDXK	6.25E-04	0.71

** Markers showing a genome-wide significance.

^a^ Chromosome number assigned according to Phillips et al. [43].

^b^ Positions obtained from female SNP map described by Lien et al. [38].

^c^ Contig containing the marker sequence as result of BLAST alignment on www.asalbase.org.

^d^ The closest identified on the contig, according to BLASTx or BLASTn results.

^e^ p-value obtained from GRAMMAR analysis and subject to Bonferroni significance thresholds.

^f^ p-value obtained from genome wide permutation analysis.

**Fig 3:**
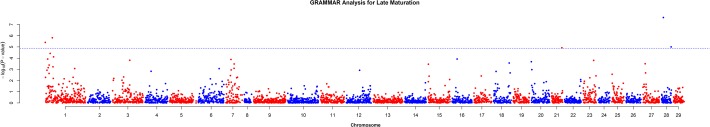
Results from GWAS for late sexual maturation. Horizontal dotted line represents the genome-wide significant threshold.

Late maturation analysis detected association with five markers (ESTNV_22894_922, ESTNV_35192_247, GCR_cBin47084_Ctg1_67, ESTNV_31055_861 and ESTNV_27268_490), which showed a genome-wide significant association with the trait according to the Bonferroni thresholds (only ESTNV_22894_922 and ESTNV_35192_247 reached genome-wide significance (p<0.05) using the permutation method), as shown in Table 3. The two most significant markers are identified as ESTNV_22894_922 and ESTNV_35192_247, and are located on chromosomes Ssa28 and Ssa01, respectively (see Fig. 3). ESTNV_22894_922 is a SNP that was assigned to sequence contig AGKD01068032 and by annotation was positioned in the coding region of FRA10AC1. ESTNV_35192_247 was assigned to sequence contig AGKD01242239 and by annotation was located in the coding region of a CPEB-associated factor maskin putative protein and assigned to sequence contig AGKD01242239.

**Table 3:** Late maturation association detected in the analysis.

SNP	Chr^a^	Position (cM)^b^	Alleles	N	Contig^c^	Harbouring gene	Nearest gene^d^	p-value GR^e^	p-value PR^f^
**ESTNV_22894_922	28	17.2	C/A	384	AGKD01068032	FRA10AC1	LGI2	2.39E-08**	0.005**
**ESTNV_35192_247	1	18.4	T/A	394	AGKD01242239	Maskin	FGFRL1	1.53E-06**	0.025**
**ESTNV_27268_490	1	0	A/T	394	AGKD01202628	Uncharacterized	TTC31	3.95E-06**	0.055
**ESTNV_31055_861	28	53.7	A/C	394	AGKD01121222	Uncharacterized	CALM1	9.95E-06**	0.075
*GCR_cBin47084_Ctg1_67	21	53.5	G/A	394	AGKD01057725	Uncharacterized	INTS6	1.20E-05**	0.08
*ESTV_20675_499	1	13.5	G/A	394	AGKD01109381	PAPL	CHRM5	3.82E-05*	0.19
*GCR_cBin27216_Ctg1_405	1	28.9	G/A	394	AGKD01052803	Uncharacterized	GARNL1	7.67E-05*	0.28
*GCR_cBin16925_Ctg1_168	16	43.9	A/G	386	AGKD01073593	Uncharacterized	PCLO	1.20E-04*	0.36
*ESTNV_26964_592	1	2.1	G/A	394	AGKD01008749	14–3–3 beta/alpha	YWHAQ	1.20E-04*	0.36
*GCR_cBin28120_Ctg1_268	7	49	C/G	394	AGKD01046830	Uncharacterized	SOX19	1.31E-04*	0.37

** Markers showing a genome-wide significance.

* Markers showing a chromosome-wide significance.

^a^ Chromosome number assigned according to Phillips et al. [43].

^b^ Positions obtained from female SNP map described by Lien et al. [38].

^c^ Contig containing the marker sequence as result of BLAST alignment on www.asalbase.org.

^d^ The closest identified gene on the contig, according to BLASTx or BLASTn results.

^e^ p-value obtained from GRAMMAR analysis and subject to Bonferroni significance thresholds.

^f^ p-value obtained from genome wide permutation analysis.

### Linkage disequilibrium (LD)

The levels of LD detected using the available markers were on average 0.22 with a median of 0.11. The estimated coefficient of the decay of LD when increasing distance (*b*_*j*_) resulting from model (1) was 6.03, indicating that, for example, the expected r^2^ for markers separated by 1 cM it is equal to 0.04. Thus, our results show that the high level of LD at shorter distances declines rapidly as distance increases (S1 Fig.). These levels of LD might be insufficient to capture the effect of genomic regions affecting quantitative traits using LD between two markers. These results suggest that the density of the SNPs used here should be improved in order to increase the power to detect association between traits and markers.

## Discussion

GWAS was able to identify at least one marker with a chromosome-wide significant association for each trait of interest. Especially surprising was the low number of markers with a significant association with growth in Atlantic salmon. We only detected one marker (GCR_cBin15343_Ctg1_36, located on Ssa13) showing a chromosome-wide significance. On the other hand, GWAS results for grilsing found five markers in genome-wide significant association with the trait (ESTNV_20578_482, ESTNV_36582_634, ESTNV_34243_316, GCR_cBin47052_Ctg1_234, ESTNV_15175_311), with all of them located on different chromosomes (Table 2). Similarly, late maturation analysis found association with five markers (according to the Bonferroni threshold), as shown in Table 3. Four of these SNPs are located on two chromosomes; ESTNV_22894_922 and ESTNV_31055_861 are located on Ssa28, but far apart from each other (36.5 cM), and the other pair of markers, located on Ssa01 (ESTNV_35192_247 and ESTNV_27268_490), are 18.4 cM apart according to the Atlantic salmon female linkage map [35].

### QTL analysis vs. GWAS

Previous studies seeking to identify genomic regions associated with phenotypic traits have based their studies on the use of linkage map regression methods and most of them used a relatively low number of markers. Growth QTL for instance, have been localized to most of the 29 Atlantic salmon chromosomes [21–23,39,51], and a similar situation has been observed in other salmonid species such as rainbow trout and Arctic charr [24,26,27,52]. As growth is a complex trait we expected to find numerous markers in association with it. Surprisingly our GWAS only identified one marker in association with growth; and this marker located on Ssa13 only showed a chromosome-wide significant association with the trait (Table 1). This finding was certainly unexpected and is contrary to previous analyses in terms of the number of regions linked to this complex trait. However, it agrees in part with results from our previous analysis [39], in which we found a genome-wide significant QTL on Ssa13 associated with growth in Atlantic salmon. As growth is a polygenic trait in Atlantic salmon, the different results obtained for the previous QTL analysis [39] and this GWAS in this population could be explained by some specific loci having a much greater effect in some of the individual families, but these effects on the trait are diluted at the population level where multiple loci with relatively small effect on the trait are involved.

The analysis for grilsing revealed five chromosome-wide significant associations with the trait by markers located on Ssa10, Ssa02, Ssa13, Ssa25 and Ssa12. In agreement with this finding, our previous QTL work based on linkage mapping described a suggestive QTL located on Ssa10 [34], but located in a different region of the chromosome. Chromosome Ssa10 shares homology with linkage group RT-8 (chromosome 5; Omy05 [53]) in rainbow trout, where a genome-wide significant QTL for early male sexual maturation was mapped [26,52] and also with linkage group AC-16 in Arctic charr, which contains a QTL associated with age at sexual maturation [24]. Moreover, these chromosomes (and also Ssa13) share the location of Clock genes implicated in the circadian regulation of many physiological functions including sexual maturation in salmonids [54]. In Atlantic salmon, QTL located on Ssa10 and Ssa12 were recently associated with precocious parr maturation [25]. To our knowledge no QTL associated with sexual maturation have been identified in Ssa02; however, several QTL related to growth have been described on this chromosome [21–23,39,51].

GWAS of late maturation allowed us to identify five markers (located on Ssa28, Ssa01 and Ssa21) showing a genome-wide significant association with the trait, along with five markers associated with the trait at a chromosome-wide level, as shown in Table 3. Previous studies analysing sexual maturation related traits in other salmonid species have identified QTL in similar regions. In rainbow trout for example, a genome-wide significant QTL associated with early maturation was found on linkage group RT-17 (Omy20 [53]), which shares homology with Ssa28 [26], and also chromosome-wide significant QTL linked to developmental rates [52]. In addition to QTL found on Ssa28, previous studies have described QTL on Ssa01 and its equivalent in other salmonid species. For instance, a QTL for age at sexual maturation in Arctic charr was described in linkage group AC-9, that shares homology with Ssa01 [32] and also a QTL for condition factor [24]. In the case of Ssa21, we recently described QTL for grilsing located in the same chromosome; however, in this analysis the QTL was detected for late maturation, which suggests that these regions controlling sexual development contain genes that are involved in both processes. QTL on this chromosome have been described for Atlantic salmon related to adult maturation [25] and in rainbow trout for early maturation [26].

Evidence based on current literature suggests that regions controlling sexual maturation are at least partially conserved within rainbow trout and Arctic charr [24,26,29]. However, the genetic architecture of this trait in Atlantic salmon still seems unresolved. Our previous analysis in Atlantic salmon detected significant QTL located on Ssa21 and Ssa18 for grilsing and late maturation, respectively, but did not detect QTL on other chromosomes [34]. Pedersen et al. [25] recently identified QTL associated with adult maturation in Ssa21 and Ssa23, but not on Ssa28. Nevertheless, they identified additional QTL that share homology with previous findings in rainbow trout and Arctic charr. Difference in populations, number of samples, markers and detection methods could explain differences found within analyses for the same species. In addition, a recent study by Johnston et al. [55] showed that the regions controlling sexual maturation in a wild population of Atlantic salmon from Northern Europe are different from those previously described in farmed fish [25, 34]. Moreover, the authors speculate that differences are due to different selection pressures in the wild and domesticated Atlantic salmon [55].

Given the different results obtained using regular QTL analysis and GWAS, we believe that such discrepancies could be explained by the different approaches used in these analytical procedures. Standard QTL analyses make use of the amount of recombination or linkage between individuals to detect association between markers and trait, which makes it a powerful method when using a low number of markers (e.g., microsatellites). On the other hand, the statistical power of GWAS is a function of sample size, effect size and marker allele frequency [56], and depends on the level of LD between the genetic markers to detect association. That being said, the relatively low power of detection observed in our analysis of growth (days to 5 kg) could be attributed to the number of markers and samples analysed [57]. Recent studies [55,57] using the same SNP chip to analyse traits in Atlantic salmon showed that the levels of LD are not sufficiently high to capture all of the genetic variation within the dataset, even with a larger sample size. A similar result regarding low values of LD between adjacent markers was found in our analyzed data (S1 Fig.), suggesting that a higher density SNP panel will be needed to fully exploit LD in GWAS.

### Candidate genes

The only marker that showed a significant association with growth in terms of days to 5 kg (chromosome-wide) was GCR_cBin15343_Ctg1_36, which is situated on an uncharacterized gene on Ssa13. Within 10 kb upstream of the location of the SNP we found MAGI-1 (membrane-associated guanylate kinase, WW and PDZ domain-containing protein 1), a protein related to cell-cell contact such as neuronal synaptic and epithelial tight junctions [58]. Another marker, which did not reach chromosome-wide level significance (p < 2.23e-4 for Ssa04), was ESTV_20925_1105, which is located in NPM1 (Nucleophosmin 1) on Ssa04. NPM1 encodes a multifunctional nucleolar phosphoprotein that plays a crucial role in the control of various aspects of cell growth and homeostasis and has been shown to be involved in the growth process of cattle [59].

Candidate genes linked to markers associated with grilsing are numerous due to the level of significance reached by the markers in association with the trait, as shown in Table 2. Marker ESTNV_20578_482 is located in an E2F transcription factor gene. E2F genes are downstream effectors of the retinoblastoma protein (pRB) pathway, and are required for the regulation of numerous genes essential for functions such as DNA replication, cell cycle progression, DNA damage repair, apoptosis, differentiation and development [60–62]. Additionally, nearby the location of this marker we found a subunit of the CCR4-NOT transcription complex, which interacts with Nanos and is essential for male germ cell development in mouse [63]. The following marker is ESTNV_36582_634, and is located in a malate dehydrogenase (MDH) gene. Differences in the enzymatic activity of MDH have been associated with developmental rates in salmonids [64,65]. Marker ESTNV_34243_316 is located in a PQ loop repeat containing protein; however, its function remains unknown.

Analysis of late maturation identified several significantly associated markers and consequently several gene candidates were identified. The most significant marker ESTNV_22894_922, assigned to Ssa28, is located in a novel gene called FRA10AC1. This gene is found within the rare FRA10A folate-sensitive fragile site, and even though its function remains unknown, it is known that in humans the 5' UTR of this gene is part of a CpG island that contains a tandem CGG repeat region (normally of 8–14 repeats but over 200 repeats when expanded) [66]. The expansion of repeats in the fragile sites results in hyper-methylation, silencing the underlying gene leading to the fragile site expression. Fragile sites have frequently been linked to mental retardation in humans [67], but are also associated with a possible role in genome reorganization due to their presence in chromosomal regions involved in rearrangements during evolution in vertebrates [68]. The second most significant marker ESTNV_35192_247 assigned to Ssa01 is located in the coding region of maskin, a CPEB-interacting protein involved in the repression of translation by its interaction with CPEB (the protein in charge of polyadenylation of mRNAs) and mainly associated with the control of oocyte maturation in *Xenopus* by repression and de-repression of mRNAs [69]. Orthologs of both FRA10AC1 and maskin are present in the Atlantic salmon genome; however, their function remains unknown. Hence further analyses of these genes are required in order to determine their possible roles in sexual maturation.

The other three SNPs that showed a genome-wide significant association with late maturation were located in uncharacterized genes (Table 3). However, the publicly available Atlantic salmon genome database (www.asalbase.org) allowed us to identify nearby genes as TTC31, DNAAF2 and INTS6 for markers ESTNV_27268_490, ESTNV_31055_861 and GCR_cBin47084_Ctg1_67, respectively.

### Potential implications for selective breeding

Most economically important traits like growth and sexual maturation are quantitative, complex traits affected by several genes [35,70]. Our GWAS failed to uncover a large number of genomic regions significantly associated with growth. A different situation was observed in the analysis of late maturation and grilsing, for which we were able to detect genome-wide significant associations. An important point to consider is the fact that markers (and their genomic regions) found in association with growth were different from those related to age at sexual maturation. This is worth noting considering that significant phenotypic and genetic correlations between growth (as estimated by body weight) and early sexual maturation in Atlantic salmon have been reported previously [13,15,71], and also taking into account that an association between growth and sexual maturation has been described in other salmonid species [24,26,28]. In Atlantic salmon, however, the link between these two traits is not conclusive, and increasing evidence indicates that growth and sexual maturation are controlled by separate regions of the genome [34,39], which also agrees with the results of this study in which the SNPs controlling growth (days to 5 kg) and sexual maturation were independent of one another. Nevertheless, due to the complexity of these traits, it is possible that regions controlling growth and sexual maturation overlap at some point in the genome but have a small effect on the trait. Pedersen et al. [25] recently found QTL for precocious parr and adult maturation in several Atlantic salmon chromosomes that have been previously shown to contain growth QTL, suggesting an interaction of certain genes in both developmental processes.

The estimated levels of heritability calculated using a pedigree-based relationship matrix, for grilsing and late sexual maturation in this population are low: grilse h^2^ = 0.09, late maturation h^2^ = 0.11 (results not shown), but in agreement with current literature levels of heritability for age at sexual maturity traits in Atlantic salmon (0.04 to 0.17 as reviewed by Garcia de Leaniz et al. [11]). Heritability of growth (days to 5 kg) on the other hand, was found to be considerably higher (h^2^ = 0.2), also in agreement with previous findings which estimated heritabilities for growth rate and body weight to range from 0.04–0.26 and 0.05–0.44, respectively [11]. Thus, selection for these traits is possible and it has been ongoing since the early 1970s [3]. Selective breeding programs have been effective in increasing body size while also controlling undesired early sexual maturity in farmed fish [2], suggesting that the main genomic regions controlling these traits are different. The use of molecular marker information could be a valuable tool to improve conventional broodstock selection programs by the identification of specific alleles that could then be screened for and introduced into selected lines.

## Conclusions

This GWAS aimed to identify genomic regions associated with growth and sexual maturation. Our previous results using a standard QTL method based on linkage mapping provided us with numerous QTL; however, the GWAS detected a lower number of markers with a highly significant association. From a biological point of view, most of the candidate genes linked to markers associated with a particular trait play a role in metabolic or developmental processes; however, it would be premature to assign an important role in the control of the trait that they were associated. Further studies, using a SNP array with a higher density, are required in order to determine if these SNPs and their affected alleles could be a valuable addition to broodstock selection programs.

## Supporting Information

S1 Fig Extent and decay of linkage disequilibrium (LD) with distance. (DOCX)
