# Supplementary material for: Genome-Wide Association Study (GWAS) for Growth Rate and Age at Sexual Maturation in Atlantic Salmon (Salmo salar)
Source: PLoS One. 2015 Mar 10;10(3):e0119730. doi: 10.1371/journal.pone.0119730 (PMC4355585; doi:10.1371/journal.pone.0119730)
Supplement: S1 Fig — (DOCX) [file pone.0119730.s001.docx]

**S1_Figure.doc Extent and decay of linkage disequilibrium (LD) with distance**


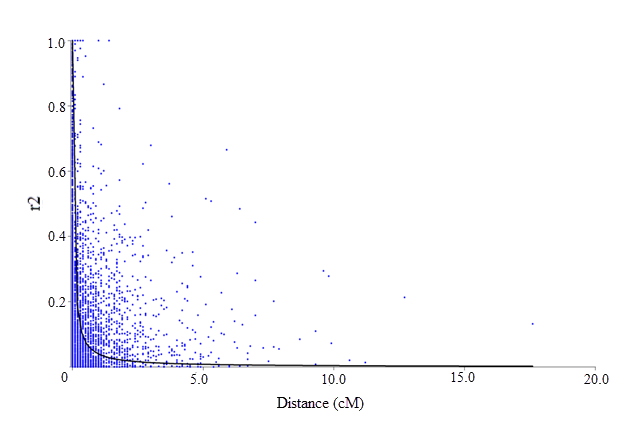


LD = *1 / (1 + 4*6.03*1) = 0.04*

Expected r2 for markers separated by 1 cM = 0.04.

LD = *1 / (1 + 4*6.03*0.1) = 0.29*

Expected r2 for markers separated by 0.1 cM = 0.29
